# Rapid and stable changes in maturation-related phenotypes of the adult hippocampal neurons by electroconvulsive treatment

**DOI:** 10.1186/s13041-017-0288-9

**Published:** 2017-03-02

**Authors:** Yuki Imoto, Eri Segi-Nishida, Hidenori Suzuki, Katsunori Kobayashi

**Affiliations:** 1 Department of Physiological Chemistry, Graduate School of Pharmaceutical Sciences, Kyoto University, Kyoto, Japan; 2 Center for Integrative Education in Pharmacy and Pharmaceutical Sciences, Graduate School of Pharmaceutical Sciences, Kyoto University, Kyoto, Japan; 3 Department of Biological Science and Technology, Faculty of Industrial Science and Technology, Tokyo University of Science, Katsushika-ku, Tokyo, Japan; 4 Department of Pharmacology, Graduate School of Medicine, Nippon Medical School, Sendagi, Bunkyō, Tokyo, Japan; 5 Japan Science and Technology Agency, Core Research for Evolutional Science and Technology, Saitama, Japan

**Keywords:** Antidepressant, Electroconvulsive seizure, Hippocampus, Maturation, Granule cell

## Abstract

Electroconvulsive therapy (ECT) is a highly effective and fast-acting treatment for depression. Despite a long history of clinical use, its mechanism of action remains poorly understood. Recently, a novel cellular mechanism of antidepressant action has been proposed: the phenotype of mature brain neurons is transformed to immature-like one by antidepressant drug treatments. We show here that electroconvulsive stimulation (ECS), an animal model of ECT, causes profound changes in maturation-related phenotypes of neurons in the hippocampal dentate gyrus of adult mice. Single ECS immediately reduced expression of mature neuronal markers in almost entire population of dentate granule cells. After ECS treatments, granule cells showed some of physiological properties characteristic of immature granule cells such as higher somatic intrinsic excitability and smaller frequency facilitation at the detate-to-CA3 synapse. The rapid downregulation of maturation markers was suppressed by antagonizing glutamate NMDA receptors, but not by perturbing the serotonergic system. While single ECS caused short-lasting effects, repeated ECS induced stable changes in the maturation-related phenotypes lasting more than 2 weeks along with enhancement of synaptic excitation of granule cells. Augmentation of synaptic inhibition or blockade of NMDA receptors after repeated ECS facilitated regaining the initial mature phenotype, suggesting a role for endogenous neuronal excitation in maintaining the altered maturation-related phenotype probably via NMDA receptor activation. These results suggest that brief neuronal activation by ECS induces “dematuration” of the mature granule cells and that enhanced endogenous excitability is likely to support maintenance of such a demature state. The global increase in neuronal excitability accompanying this process may be relevant to the high efficacy of ECT.

**Electronic supplementary material:**

The online version of this article (doi:10.1186/s13041-017-0288-9) contains supplementary material, which is available to authorized users.

## Background

Granule cells (GCs) in the hippocampal dentate gyrus (DG) have been implicated in the pathophysiological mechanisms of neuropsychiatric disorders including depression and schizophrenia, and have been suggested to be an important target for both pharmacological and physical therapeutic treatments [1–6]. We have recently demonstrated distinct changes in the molecular and physiological phenotypes of GCs as a candidate cellular mechanism of action of a selective serotonin reuptake inhibitor (SSRI): The SSRI fluoxetine can transform several mature features of GCs in the adult mouse DG [7]. The fluoxetine treatment strongly reduced expression of mature GC markers, induced active somatic membrane properties resembling immature GCs, and decreased short-term plasticity at the dentate-to-CA3 synaptic connection that characterizes the mature dentate-to-CA3 signal transmission. These changes cannot be explained simply by an increase in newly generated immature GCs, but are most likely characterized as “dematuration” of mature GCs [7].

These changes in the maturation-related phenotypes of GCs were not observed after short-term treatment of fluoxetine, and the efficacy of fluoxetine is quite variable among individual mice [7, 8]. Such unstable and unpredictable nature of the effects of fluoxetine well mimics common clinical observations in antidepressant medication such as delayed emergence of therapeutic effects and treatment resistance [9]. In a mouse model of depression and anxiety, the changes in the maturation-related phenotypes of GCs can be induced at a serum concentration of fluoxetine close to that seen in patients during fluoxetine medication [10], suggesting clinical relevance of these changes in GCs. Therefore, we proposed that modifications of maturation-related phenotypes in mature neurons may cause some beneficial or therapeutic effects of SSRIs by reinstating cellular functions of immature neurons. This proposal has been supported by demonstration of similar SSRI-induced changes in the neuronal maturation status in other brain regions [11, 12].

In addition to antidepressant drugs, electroconvulsive therapy (ECT) has been used to treat depression. ECT was originally devised for treating psychosis in the 1930s [13] and is currently considered as the most effective treatment for depression [14]. ECT has fast-acting antidepressant effects and is effective in most of medication-resistant patients. Despite a long history of clinical use, the mechanism of action of ECT still remains poorly understood. In particular, it is unknown how distinct types of treatments, antidepressant drug medication and ECT, converge on the same antidepressant action. In rodents, ECT-like stimulation has been shown to reduce immunoreactivity for the calcium binding protein calbindin in the DG [15, 16]. Calbindin has been considered as a marker for mature GCs, because its expression is not seen in newly generated GCs at the cell age of 10 days or less and is established in about 4 weeks [17]. A reduction of calbindin expression is one of characteristic features of demature GCs in SSRI-treated mice. Therefore, it is possible that ECT-like stimulation can also induce dematuration of GCs, possibly in a more rapid and consistent manner than SSRI treatments. To test this hypothesis, in the present study, we examined how electroconvulsive stimulation (ECS), an animal model of ECT, regulates maturation-related phenotypes of the mature GCs in the adult mouse DG.

## Results

### Downregulation of mature granule cell markers by ECS

**Fig. 1:**
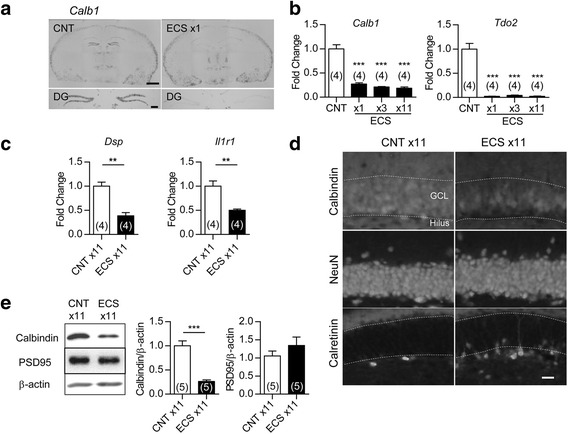
Alteration of GC maturation stage markers in the DG after ECS. **a** *,* Representative images of *in situ* hybridization of *Calb1* at 6 h after single ECS or sham (CNT) treatment. Scale bars: upper 1 mm, lower 200 μm. **b**, The relative expression of *Calb1* and *Tdo2* at 6 h after the indicated number of ECS (Dunnett’s test following one-way ANOVA: *F*_(3,12)_ = 69.72, *** *P* < 0.001 for *Calb1*, *F*_(3,12)_ = 67.15, *** *P* < 0.001 for *Tdo2*). **c**, The relative expression of *Dsp* and *Il1r1* after 11 ECS sessions (*t*_(6)_ = 5.733, ** *P* < 0.01 for *Dsp*, *t*_(6)_ = 4.418, ** *P* < 0.01 for *Il1r1*). **d**, Representative images of immunoreactivity for NeuN, calbindin, and calretinin in DG at 24 h after 11 ECS sessions. GCL: granule cell layer. Scale bar: 20 μm. **e**, Immunoblot detections of reduced calbindin D-28 K expression (*t*_(8)_ = 6.899, *** *P* < 0.001) and intact PSD-95 expression in DG (*t*_(8)_ = 1.082, *P* = 0.31). The n number is given in graph. Data are presented as means ± SEM

**Fig. 2:**
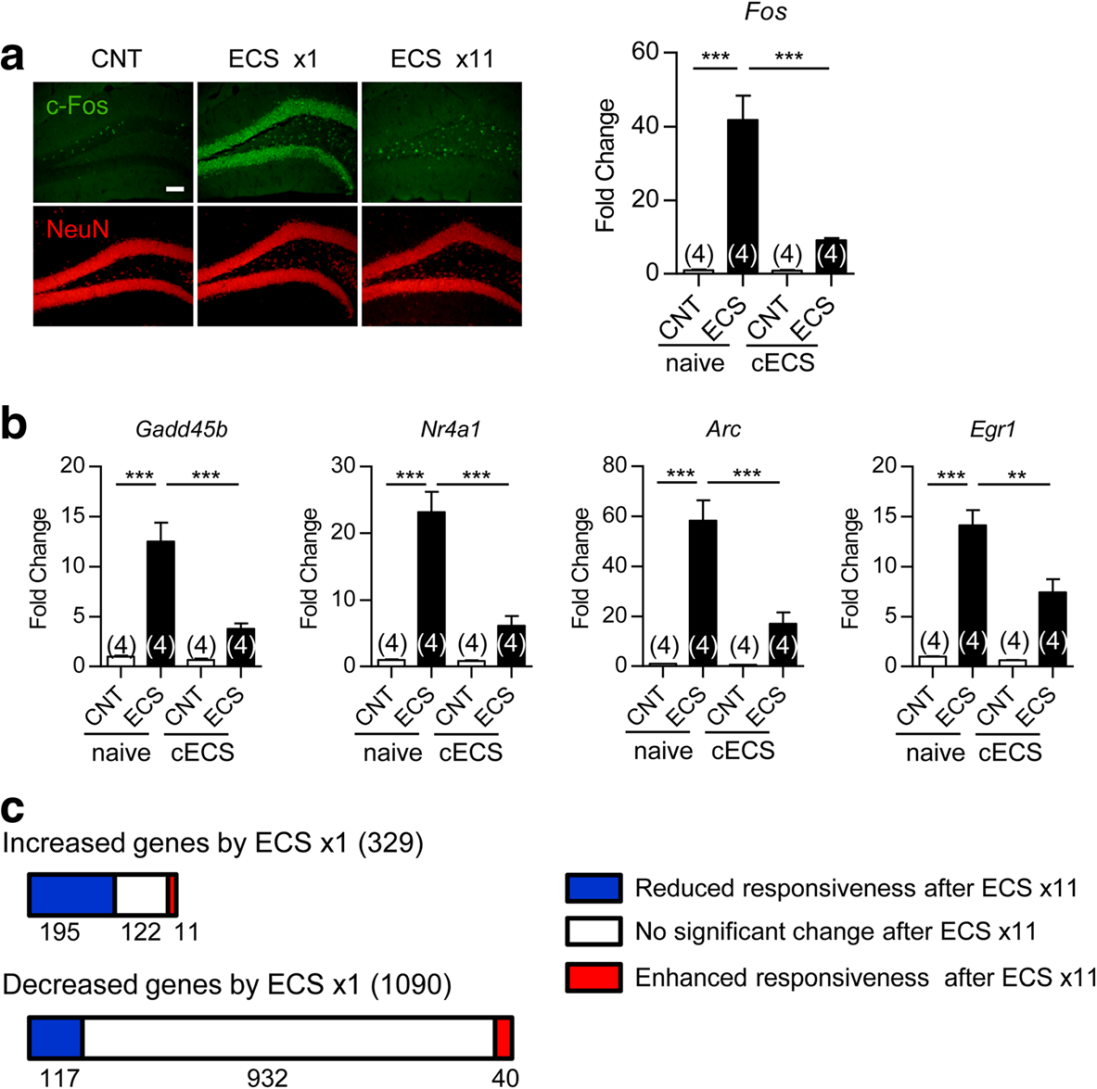
Altered stimulus-induced gene expression in DG after repeated ECS. **a**, Left: representative images of immunoreactivity for c-fos and NeuN at 2 h after the last ECS. Scale bar, 100 μm. Right: the sham (CNT) treatment or ECS was applied to naive mice or mice treated with 10 times of chronic ECS (cECS). The expression of *Fos* at 1 h after the last treatment was examined (Tukey’s test following one-way ANOVA: *F*_(3,12)_ = 35.77, *** *P* < 0.001). **b**, The relative expression of other IEGs (*Gadd45b*, *Nr4a1*, *Arc*, and *Egr1*) mRNA at 1 h after the last ECS or control treatment (Tukey’s test following one-way ANOVA: *F*_(3,12)_ = 31.16, *** *P* < 0.001 for *Gadd45b*, *F*_(3,12)_ = 38.36, *** *P* < 0.001 for *Nr4a1*, *F*_(3,12)_ = 33.39, *** *P* < 0.001 for *Arc*, *F*_(3,12)_ = 39.82, ** *P* < 0.01, *** *P* < 0.001 for *Egr1*). **c**, The number of genes that showed a significant increase or decrease by a single ECS-treatment is represented in bars. The three groups that were categorized according to expression change by repeated ECS were color-coded. The n number is given in graph. Data are presented as means ± SEM

The matured state of GCs is characterized by several distinct molecular and physiological features [18, 19], and chronic SSRI treatment can change some of such features to immature-like ones [7, 8, 10, 20]. We first examined the effect of ECS on the expression of the mature GC marker calbindin in adult mice and found that expression of the gene encoding calbindin (*Calb1*) in the DG was entirely reduced by a single ECS (Fig. 1a), which is consistent with the previous observations of the reduced calbindin-like immunoreactivity after ECS [15, 16]. A single and repeated ECS (11 times over a period of 3 weeks) reduced the expression of *Calb1* and that of the gene encoding tryptophan 2,3-dioxygenase (*Tdo2*), another mature GC marker [7, 21], at 6 h after the stimulation (Fig. 1b). After repeated ECS, the gene expression of desmoplakin (*Dsp*) and interleukin-1 receptor type 1 (*Il1r1*), which show developmental expression profiles similar to those of *Calb1* and *Tdo2* [7], was also reduced (Fig. 1c). The global reduction of calbindin expression after repeated ECS was confirmed at the protein level (Fig. 1d, e). In contrast, the same treatment did not affect the expression of the neuronal marker NeuN (Fig. 1d) or PSD-95 (Fig. 1e), which excludes the possibility that ECS caused non-specific damage on GCs. Repeated ECS upregulated the expression of calretinin, a marker of immature GCs at the early post-mitotic stage, in the subgranular zone, probably via increased adult neurogenesis. The majority of the NeuN-positive and calbindin-negative GCs in the DG did not express calretinin after ECS (Fig. 1d). We next examined the effect of ECS on the stimulus-induced expression of immediate early genes (IEGs), which is an index of the maturity of activity-dependent neuronal responsiveness in vivo [18, 19, 22]. While a single ECS induced robust expression of the IEG c-fos in the majority of GCs, this c-fos expression in GCs was strongly suppressed after repeated ECS (Fig. 2a). Repeated ECS significantly suppressed robust induction of other IEGs, *Gadd45b, Nr4a1, Arc,* and *Egr1*, in the DG at 1 h after the stimulation (Fig. 2b). We further compared gene expression profiles at 1 h after the stimulation between single ECS-treated and repeated ECS-treated DGs. Among genes that showed significant changes by a single ECS treatment (329 increased genes and 1,090 decreased genes), more than 300 genes (195 increased genes and 117 decreased genes) showed reduced responsiveness after repeated ECS treatments (Fig. 2c). Functional gene ontology classification revealed that the genes that showed reduced responsiveness after repeated ECS are strongly associated with cellular responses to a variety of stimuli including cAMP, hormones, and cytokines as well as regulation of transcription (Additional file 1: Table S1-S2).

### ECS-treated granule cells partially exhibit physiological properties resembling immature neurons

**Fig. 3:**
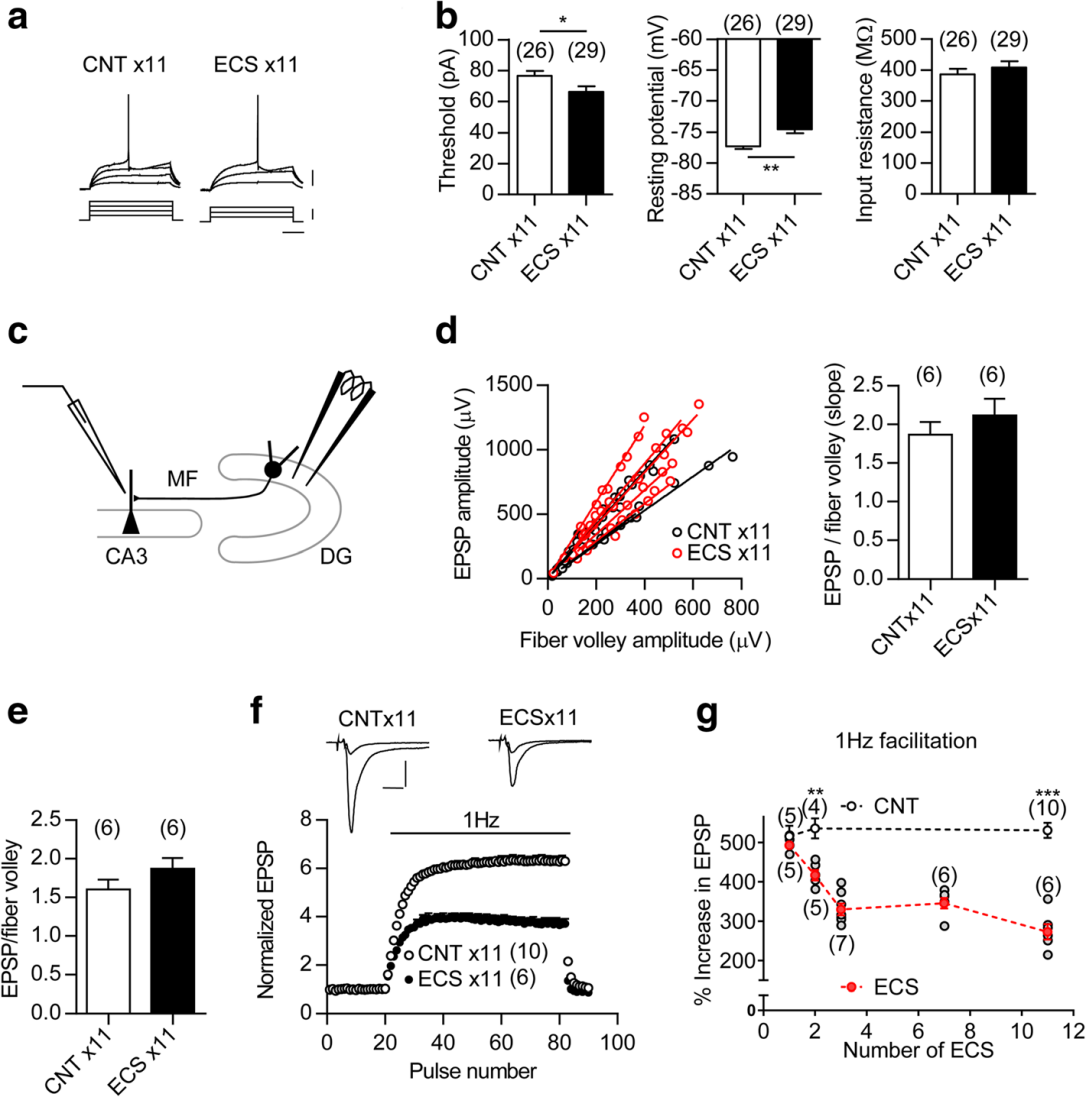
Immature-like functional properties of GC soma and output synapse after ECS treatment. **a**, Membrane potential changes (upper) induced by depolarizing currents (lower) in GCs. Scale bars: 100 ms, 20 mV, 40 pA. **b**, Left: the threshold current intensity required to evoke a single spike (*t*_(53)_ = 2.157, * *P* =0.0356). Center: resting membrane potential (*t*_(48)_ = 3.475, ** *I* = 0.0011, Student’s t-test with Welch’s correction). Right: input resistance. The number (n) represents the number of cells. **c**, A diagram showing the electrode arrangement for recording field EPSPs at the MF-CA3 synapse. **d**, No significant change in the input-output relationship at the MF synapse. Left: The relationship between field EPSP and fiber volley amplitude was examined by changing stimulus intensities. Right: The slope value of regression line is shown (*t*_(10)_ = 0.9684, *P* = 0.3557). The n number represents the number of slices. **e**, No significant change in MF field EPSP to fiber volley ratios at the baseline stimulus intensity (*t*_(10)_ = 0.9324, *P* = 0.3731). **f**, Reduction of 1-Hz frequency facilitation at the MF synapse after repeated ECS. Inset: sample recordings of MF field EPSPs during baseline and 1-Hz stimulation. Scale bars: 10 ms, 0.5 mV. **g**, Reduction of frequency facilitation at the MF synapse after ECS repeated twice or more times (ECS × 2, *t*_(7)_ = 4.364, ** *P* = 0.0033; ECS × 11, *t*_(14)_ = 8.832, *** *P* < 0.001). Both individual (grey) and mean data (red) are shown for ECS-treated groups. The number (n) is shown in the graph. Data are presented as means ± SEM

Above results demonstrate that ECS strongly downregulates several markers for mature GCs, which resembles the effects of chronic SSRI treatments on the dentate GCs [7, 10, 20]. We then examined the electrophysiological properties of ECS-treated GCs. Immature GCs show higher somatic excitability, higher input resistance, and more depolarized resting membrane potentials than mature GCs [23, 24]. Repeated ECS increased the somatic excitability of GCs and reduced their resting membrane potentials, but had no significant effect on input resistance (Fig. 3a, b). We next analyzed the synaptic transmission between the GC axon mossy fibers (MFs) and the CA3 pyramidal cells (Fig. 3c). Although the MF synapse in adult mice is characterized by strong frequency facilitation, a form of presynaptic short-term plasticity, the immature MF synapse in juvenile mice exhibits greatly reduced frequency facilitation [7, 25]. While repeated ECS had no significant effect on the basal synaptic efficacy (Fig. 3d, e), it consistently reduced the frequency facilitation of excitatory postsynaptic potentials (EPSPs) at the MF synapse to a juvenile level (Fig. 3f, g). Therefore, ECS-treated GCs showed some of physiological properties characteristic of immature GCs.

### ECS-treated mice share gene expression changes in DG with SSRI-treated mice and genetically modified mice with altered GC maturation

**Fig. 4:**
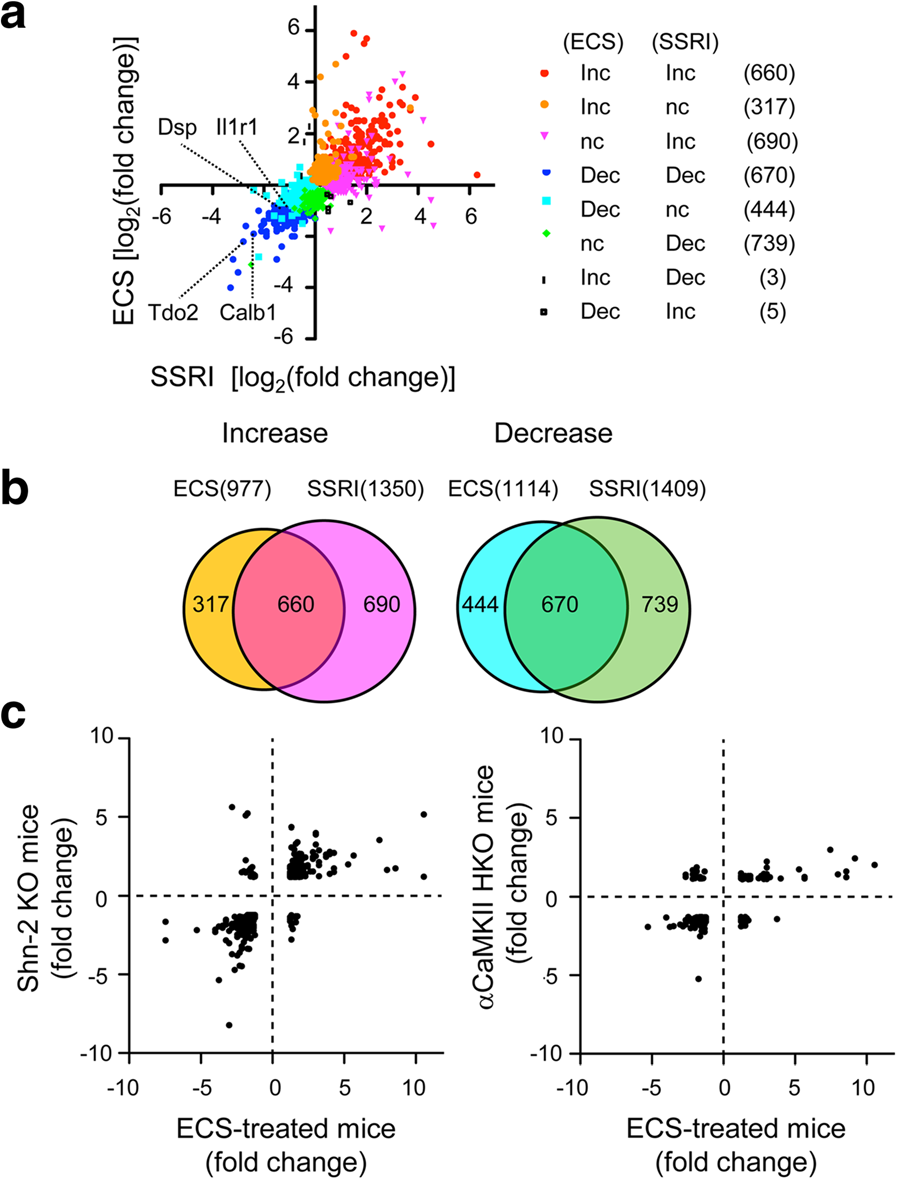
Shared gene expression changes in repeated ECS-treated DG, chronic SSRI-treated DG and hippocampus of mutant mice with altered DG maturation. **a**, Scatter correlation graph of gene expression changes [log_2_(fold-change)] between repeated ECS-treated and chronic SSRI-treated DGs. The gene probes (3,531) that showed significant changes by either ECS- or SSRI-treatment in microarray analysis are shown. The eight groups that were categorized according to expression change by ECS and/or SSRI treatment were color-coded. **b**, The number of genes that showed significant expression changes by either ECS- or SSRI-treatment are represented in a Venn diagram. The color corresponds to that in (**a**). **c**, Scatter correlation graph illustrating the fold change for gene expression levels in the DG from repeated ECS-treated mice, the hippocampus from Shn-2 KO mice (558 gene probes, left), and the hippocampus from αCaMKII hetero KO mice (190 gene probes, right). The genes that showed statistically significant and more than 1.2-fold changes in both the DG from ECS-treatment mice and the hippocampus from mutant mice were selected

The above results suggested that ECS and SSRI have similar effects on the phenotype of GCs. To further address this point, we next compared a gene expression profile between chronic SSRI- and repeated ECS-treated DGs. Among 3,531 genes that showed significant changes by either ECS- or SSRI-treatment, 660 genes showed significant increases and 670 genes showed significant decreases by both ECS- and SSRI-treatment (Fig. 4a, b). Significant correlation was identified in the gene expression change between ECS-treated DG and SSRI-treatment DG (*r* = 0.76, *p* < 0.0001). Functional gene ontology classification revealed that the genes that showed significant increases by both treatments are strongly associated with cholesterol metabolic process, nervous system development, and neuron projection development (Table 1). The genes that showed significant decreases by both treatments also showed a strong association with nervous system development (Table 2). In contrast, the genes that were not commonly changed are less associated with neuronal functions (Additional file 1: Table S3-S6). These results suggest that repeated ECS and chronic SSRI induce a similar change in the phenotypes of GCs, especially in those related to neuronal functions and development, in the adult DG.

**Table 1:** Functional classification on the basis of the gene ontology (GO) terms

Increased change by both ECS- and SSRI-treatment (660 probes)			
GO number	GO term	No.	*P* value
GO:0008203	cholesterol metabolic process	16	9.8E-09
GO:0016126	sterol biosynthetic process	10	1.1E-08
GO:0008202	steroid metabolic process	15	3.5E-08
GO:0006695	cholesterol biosynthetic process	9	1.3E-06
GO:0006694	steroid biosynthetic process	11	4.4E-06
GO:0007399	nervous system development	26	2.1E-05
GO:0010977	negative regulation of neuron projection development	10	4.1E-05
GO:0061564	axon development	5	5.3E-05
GO:0010976	positive regulation of neuron projection development	14	7.6E-05
GO:0042493	response to drug	24	1.2E-04
GO:0051591	response to cAMP	9	1.4E-04
GO:0009790	embryo development	10	2.2E-04
GO:0006950	response to stress	7	2.3E-04
GO:0001649	osteoblast differentiation	12	2.4E-04
GO:0031668	cellular response to extracellular stimulus	6	2.8E-04
GO:0034097	response to cytokine	10	3.5E-04
GO:0007568	aging	15	3.6E-04
GO:0030154	cell differentiation	38	3.8E-04
GO:0009968	negative regulation of signal transduction	8	4.4E-04
GO:0007155	cell adhesion	27	4.9E-04
GO:0006629	lipid metabolic process	26	5.1E-04
GO:0007565	female pregnancy	10	5.4E-04
GO:0043065	positive regulation of apoptotic process	21	6.1E-04
GO:0043627	response to estrogen	9	7.6E-04
GO:0007179	transforming growth factor beta receptor signaling pathway	9	8.3E-04
GO:0043407	negative regulation of MAP kinase activity	7	8.4E-04
GO:2001237	negative regulation of extrinsic apoptotic signaling pathway	7	9.5E-04
GO:0010628	positive regulation of gene expression	23	9.6E-04

The gene probes (660 probes) showed significantly increased change by both ECS- and SSRI-treatment were selected. *P*-value was calculated by a modified Fisher’s exact test. Highly associated gene ontology terms (*P* < 0.001) were represented

**Table 2:** Functional classification on the basis of the gene ontology (GO) terms

Decreased change by both ECS- and SSRI-treatment (670 probes)			
GO number	GO term	No.	*P* value
GO:0007399	nervous system development	26	5.12E-05
GO:0045944	positive regulation of transcription from RNA polymerase II promoter	50	7.54E-05
GO:0016310	phosphorylation	33	4.24E-04
GO:0007169	transmembrane receptor protein tyrosine kinase signaling pathway	11	4.45E-04
GO:0070498	interleukin-1-mediated signaling pathway	5	4.63E-04
GO:0008284	positive regulation of cell proliferation	30	5.84E-04
GO:0071277	cellular response to calcium ion	8	6.06E-04
GO:0006468	protein phosphorylation	31	7.05E-04
GO:0048511	rhythmic process	12	8.55E-04

The gene probes (670 probes) showed significantly decreased change by both ECS- and SSRI-treatment were selected. *P*-value was calculated by a modified Fisher’s exact test. Highly associated gene ontology terms (*P* < 0.001) were represented

We have previously shown that adult dentate GCs stay in an immature-like state in mice heterozygous for α-calcium/calmodulin-dependent protein kinase II (α-CaMKII hetero KO) [2] and in Schnurri-2 knockout (Shn-2 KO) mice [26]. To further characterize the phenotype of ECS-treated GCs, we next compared the pattern of the gene expression change in the ECS-treated DG with that in the hippocampus from these mutant mice. The genes that showed statistically significant and more than 1.2-fold changes were plotted in Fig. 4c. Significant correlation was identified in the gene expression change between ECS-treated DG and Shn-2 KO hippocampus (*r* = 0.65, *p* < 0.0001) and between ECS-treated DG and α-CaMKII hetero KO hippocampus (*r* = 0.61, *p* < 0.0001). Among the 30 genes most up- or down-regulated (15 genes each) by both ECS- and SSRI-treatment, the expression for *Calb1, Tdo2, Frzb, Batf3,* and *Grp* was commonly regulated in the hippocampus of Shn-2 KO mice and α-CaMKII hetero KO mice (Tables 3 and 4). These results suggest that the ECS-treated GCs share the phenotype with immature-like GCs of Shn-2 KO mice and α-CaMKII hetero KO mice.

**Table 3:** Most up-regulated genes by ECS treatment among genes significantly regulated by both ECS- and SSRI-treatment (Top 15 genes)

Affymetrix_ID	Gene	ECS-treated	SSRI-treated	Shn-2 KO	αCaMKII hetero KO
1427683_at	*Egr2*	5.9	1.5	n.c.	n.c.
1422134_at	*Fosb*	5.5	1.9	n.c.	n.c.
1423100_at	*Fos*	5	1.2	n.c.	n.c.
1448594_at	*Wisp1*	3.8	3.4	1.2	n.c.
1418687_at	*Arc*	3.7	2.6	n.c.	n.c.
1425671_at	*Homer1*	3.6	1.4	n.c.	0.7
1418930_at	*Cxcl10*	3.4	3.9	n.c.	n.c.
1453076_at	*Batf3*	3.4	2.5	2.4	1.1
1420720_at	*Nptx2*	3.2	3.3	n.c.	1.3
1427038_at	*Penk1*	3.1	3.4	n.c.	0.8
1417933_at	*Igfbp6*	3.1	2.9	n.c.	0.4
1436563_at	*4932441J04Rik*	3.1	1.2	0.8	n.c.
1417262_at	*Ptgs2*	3.0	1.4	n.c.	0.6
1424525_at	*Grp*	2.9	3.7	1.8	1.6
1419127_at	*Npy*	3.0	2.7	n.c.	n.c.

**Table 4:** Most down-regulated genes by ECS treatment among genes significantly regulated by both ECS- and SSRI-treatment (Top 15 genes)

Affymetrix_ID	Gene	ECS-treated	SSRI-treated	Shn-2 KO	αCaMKII hetero KO
1450803_at	*Ntf3*	−4.0	−3.3	−1.9	n.c.
1426980_s_at	*E130012A19Rik*	−3.5	−2.3	n.c.	n.c.
1419663_at	*Ogn*	−3.4	−3.0	−1.9	n.c.
1425179_at	*Shmt1*	−2.9	−1.5	−0.7	n.c.
1448424_at	*Frzb*	−2.4	−1.3	−1.1	−0.9
1419093_at	*Tdo2*	−2.2	−2.8	−3.8	−3.4
1417858_at	*Rasal1*	−2.0	−0.8	n.c.	n.c.
1452114_s_at	*Igfbp5*	−2.0	−0.9	−1.1	n.c.
1421595_at	*9630031F12Rik*	−2.0	−1.9	−1.2	−0.3
1460203_at	*Itpr1*	−1.9	−1.9	−1.0	n.c.
1448738_at	*Calb1*	−1.9	−2.4	−2.4	−0.9
1420401_a_at	*Ramp3*	−1.8	−1.9	n.c.	n.c.
1424214_at	*9130213B05Rik*	−1.7	−0.8	n.c.	0.19
1435162_at	*Prkg2*	−1.7	−0.8	−0.6	n.c.
1449571_at	*Trhr*	−1.7	−1.0	n.c.	n.c.

### Involvement of NMDA receptor-dependent signaling in the rapid downregulation of maturation markers by ECS

**Fig. 5:**
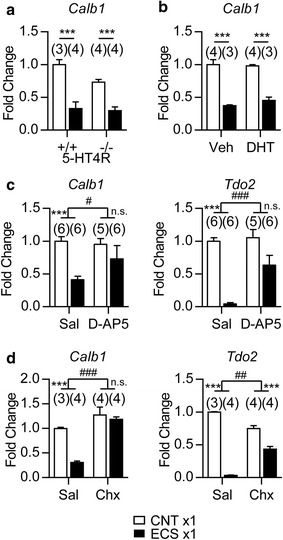
NMDA receptor activation and *de novo* protein synthesis are involved in the rapid downregulation of maturation markers by ECS. **a**, The relative expression of *Calb1* in 5-HT4R-KO (-/-) mice at 6 h after a single ECS (*** *P* < 0.001, Bonferroni’s *post hoc* test following two-way ANOVA). **b**, The relative expression of *Calb1* in mice treated with 5,7-DHT (i.c.v.) at 6 h after a single ECS (*** *P* < 0.001, Bonferroni’s *post hoc* test following two-way ANOVA). Veh: vehicle. **c**, The relative expression of *Calb1* and *Tdo2* at 6 h after a single ECS in mice treated with D-AP5 (1 μg/mouse, interaction [D-AP5 × ECS]; *F*_(1,19)_ = 7.07 for *Calb1,* # *P* < 0.05*, F*_(1,19)_ = 15.7 for *Tdo2,* ### *P* < 0.001). **d**, The relative expression of *Calb1* and *Tdo2* at 6 h after a single ECS in mice treated with cycloheximide (Chx; 200 mg/kg, i.p., interaction [cycloheximide × ECS]; *F*_(1,11)_ = 11.35 for *Calb1,* ### *P* < 0.001*, F*_(1,11)_ = 149.1 for *Tdo2,* ## *P* < 0.01). Bonferroni’s *post hoc* test following two-way ANOVA, *** *P* < 0.001, n.s., not significant. Sal: saline. The number (n) is shown in the graph. Data are presented as means ± SEM

Unlike SSRI-induced changes in the maturation-related phenotypes, which are not observed after short-term drug treatments [7], the ECS-induced changes in GCs, especially downregulation of mature GC markers, rapidly developed (Fig. 1b). We next explored the mechanism involved in this rapid effect of ECS. We first examined the involvement of the serotonin type 4 receptor (5-HT4R) in downregulation of *Calb1* expression in GCs, because this receptor plays a key role in the SSRI-induced change in the maturation-related phenotypes of GCs [7, 20]. However, neither deficiency of 5-HT4R nor a lesion of the central serotonergic neurons induced by 5,7-dihydroxytryptamine (5,7-DHT) affected the reduction of *Calb1* expression by a single ECS (Fig. 5a, b). Therefore, the serotonergic system is not required for the rapid downregulation of the maturation marker by ECS.

We then examined the involvement of an N-methyl-D-aspartate receptor (NMDAR) using D-(-)-2-amino-5-phosphonopentanoic acid (D-AP5), an NMDAR antagonist. Intracerebroventricularly injected D-AP5 attenuated the reduction of *Calb1* and *Tdo2* expression levels by a single ECS (Fig. 5c). As NMDAR activation upregulates expression of many genes in the DG, gene induction and subsequent *de novo* protein synthesis may be necessary for ECS-induced downregulation of mature GC markers. As expected, pretreatments with cycloheximide, a protein synthesis inhibitor, attenuated the reduction of *Calb1* and *Tdo2* by a single ECS (Fig. 5d). These results suggested that protein synthesis and NMDAR activation are important for the rapid downregulation of mature GC markers by ECS.

### Long-lasting phenotypic change in granule cells after repeated ECS

**Fig. 6:**
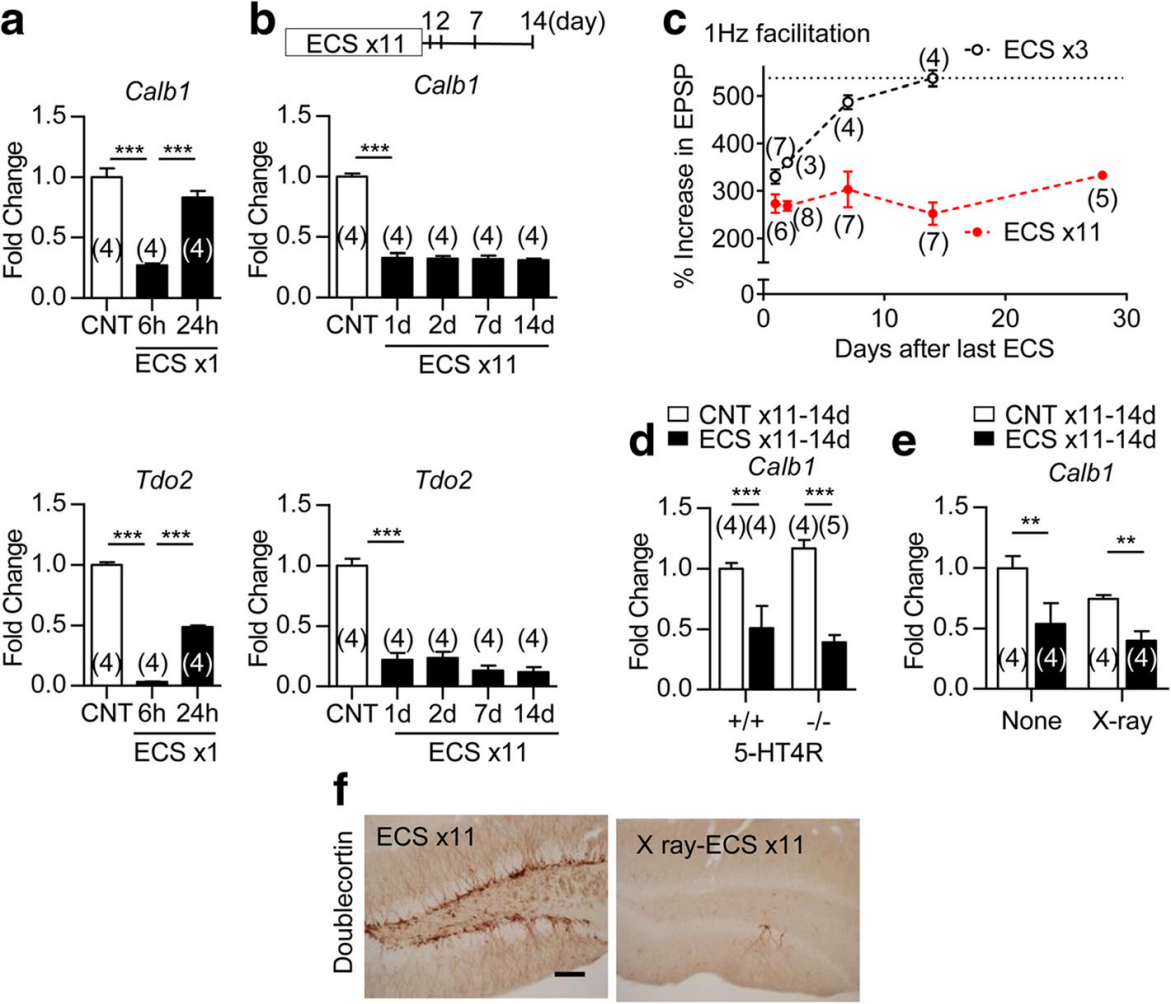
Long-lasting phenotypic change in GCs after repeated ECS. **a**, **b**, The relative expression of *Calb1* and *Tdo2* at indicated time intervals after single (**a**) or repeated (**b**) ECS (**a**, Tukey’s test following one-way ANOVA: *F*_(2,9)_ = 54.21, *** *P* < 0.001 for *Calb1*, *F*_(2,9)_ = 1048, *** *P* < 0.001 for *Tdo2,* **b**, *F*_(4,15)_ = 123, *** *P* < 0.001 for *Calb1*, *F*_(4,15)_ = 53.67, *** *P* < 0.001 for *Tdo2*). **c**, Frequency facilitation tested at various time intervals after three or 11 times of ECS. **d**, The relative expression of *Calb1* was compared between 5-HT4R-KO (-/-) mice and wild-type (+/+) mice at 14 days after 11 times of ECS (*** *P* < 0.001, Bonferroni’s *post hoc* test following two-way ANOVA). **e**, The effect of X-ray (10 Gy) on the reduction of *Calb1* at 14 days after 11 times of ECS. (** *P* < 0.01, Bonferroni’s *post hoc* test following two-way ANOVA). **f**, Representative images of immunoreactivity for doublecortin in non-irradiated (ECS × 11) or irradiated (X-ray-ECS × 11) mice at 14 days after 11 times of ECS. Scale bar: 200 μm. The number (n) is shown in the graph. Data are presented as means ± SEM

We next examined the maintenance of the change in maturation-related phenotypes of GCs after ECS. Although a single ECS robustly reduced the expression of *Calb1* and *Tdo2* in the DG at 6 h after ECS, the expression levels returned toward control levels within 24 h (Fig. 6a). In contrast, the reduction of *Calb1* and *Tdo2* expression levels was maintained at least for 14 days after repeated ECS (Fig. 6b). Similarly, while the reduction of frequency facilitation at the MF synapse was transient after 3 times of ECS, the reduction of frequency facilitation was maintained more than 28 days after 11 times of ECS (Fig. 6c). The deficiency of 5-HT4R did not affect the reduction of *Calb1* expression at 14 days after repeated ECS (Fig. 6d), indicating that 5-HT4R is not required for the long-lasting phenotypic change of GCs caused by ECS. We also examined whether the long-lasting reduction of *Calb1* expression in GCs depends on newly generated neurons. Cranial X-ray irradiation mostly depleted the staining of doublecortin, a marker for early immature GCs, even after repeated ECS (Fig. 6f). In these X-ray-irradiated mice, the reduction of *Calb1* expression was normally observed at 14 days after repeated ECS (Fig. 6e). Taken together, these results suggested that brief neuronal activation by ECS rapidly changed the maturation-related phenotypes in mature GCs, and that repeated treatments could convert the transient phenotypic change into a robust long-lasting one.

### Role of GABAergic signaling in reversal of long-lasting phenotypic change

**Fig. 7:**
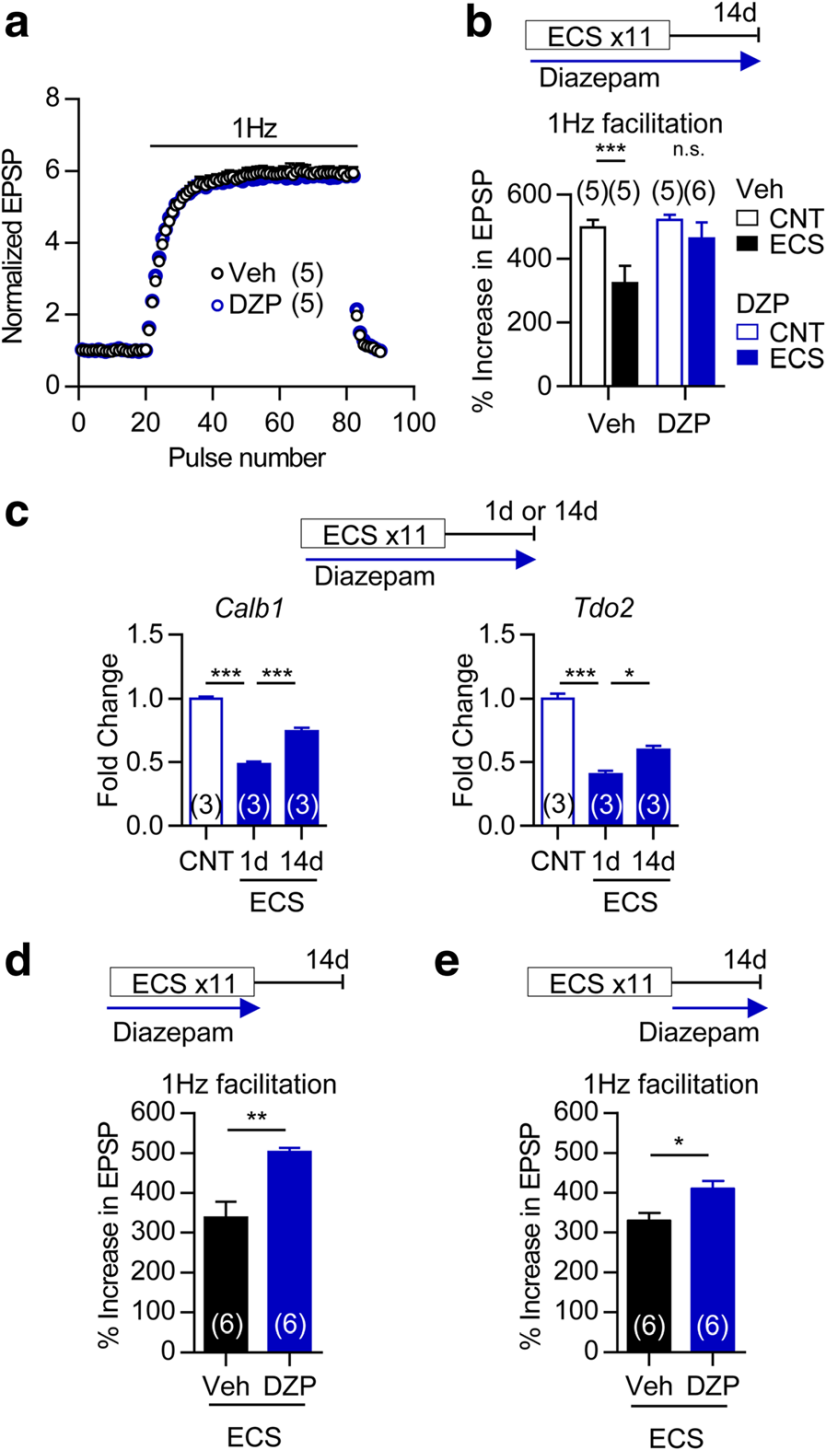
Augmentation of GABAergic signalling reverses long-lasting phenotypic change. **a**, No significant difference was seen in frequent facilitation at the MF synapse between mice treated with diazepam (DZP, 5 mg/kg) and vehicle (Veh) for 3 weeks. **b**, Frequency facilitation at 14 days after 11 times of ECS in mice treated with 5 mg/kg diazepam or vehicle (*** *P* < 0.001, Bonferroni’s test following two-way ANOVA, interaction [drug treatment × ECS]; *F*_(1,17)_ = 7.39, P = 0.0146). **c**, The relative expression of *Calb1* and *Tdo2* at 1 day or 14 days after 11 times of ECS in mice treated with 10 mg/kg diazepam (Tukey’s test following one-way ANOVA: *F*_(2,6)_ = 122.1, *** *P* < 0.001 for *Calb1*, *F*_(2,6)_ = 90.9, * *P* < 0.05, *** *P* < 0.001 for *Tdo2*). **d**, Effects of diazepam on frequency facilitation at 14 days after 11 times of ECS. Diazepam (5 mg/kg) was administered during the period of ECS treatments (*t*_(10)_ = 4.062, *P* = 0.0097). **e**, Effects of diazepam (10 mg/kg) administered after 11 times of ECS on the lasting reduction of frequency facilitation (*t*_(10)_ = 2.873, *P* = 0.0166). The number (n) is shown in the graph. Data are presented as means ± SEM

Since activation of excitatory synaptic transmission by ECS is important for the change in the maturation-related phenotype of GCs, we next examined a possibility that GABAergic inhibitory synaptic transmission could play a role in modulating this process. Diazepam, a positive allosteric modulator of GABA_A_ receptor, was administered during and/or after the ECS treatment period. Chronic diazepam treatment had no significant effect on frequency facilitation at the MF synapse in control mice (Fig. 7a), suggesting a lack of effects on the mature state of GCs. In diazepam-treated mice, however, the long-lasting reduction of frequency facilitation was attenuated at 14 days after repeated ECS (Fig. 7b), and the decreased expression of mature GC markers, *Calb1* and *Tdo2*, was significantly reversed at 14 days after repeated ECS (Fig. 7c). Diazepam applied only during the period of ECS treatments was similarly effective in attenuating the reduction of frequency facilitation at 14 days after repeated ECS (Fig. 7d). Diazepam applied only after the period of ECS treatments partially, but significantly, reversed the reduction of frequency facilitation (Fig. 7e). Thus, enhanced GABAergic inhibition prevents the establishment of the lasting changes in the maturation-related phenotype.

### Increased synaptic activation of granule cells after repeated ECS

**Fig. 8:**
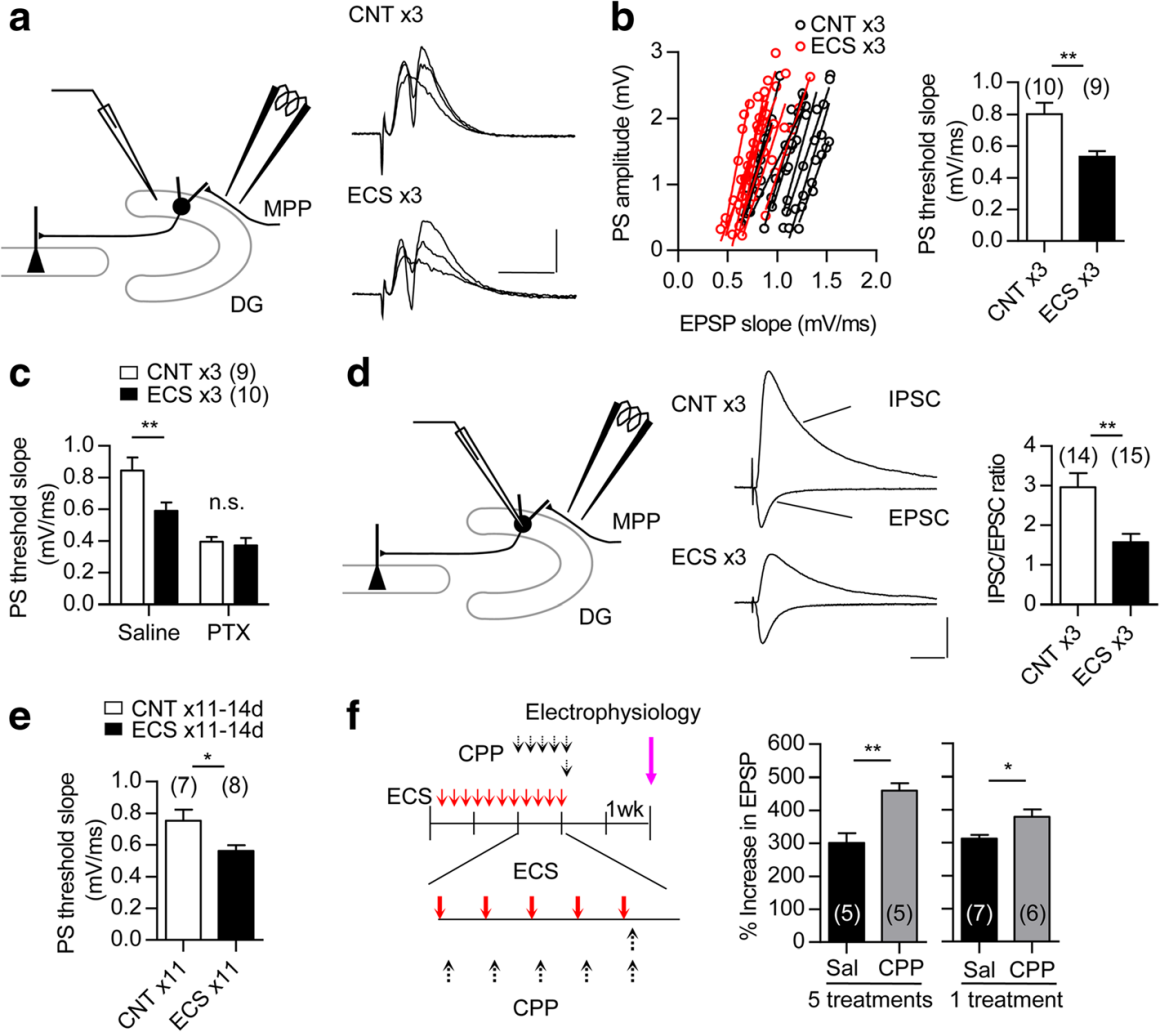
Enhanced synaptic activation in GCs after repeated ECS. **a**, Left: A diagram showing the electrode arrangement for recording GC population spikes (PSs) evoked by MPP stimulation. Right: Sample recordings of PSs evoked at three different intensities. Scale bars: 10 ms, 1 mV. **b**, Left: The relationship between field EPSP slope and PS amplitude recorded in the GC layer after three times of ECS. Right: X-intercepts of linear regression lines were measured to determine the threshold EPSP slope for evoking PS. (*t*_(13)_ = 3.356, ** *P* = 0.0052, Student’s t-test with Welch’s correction, n represents the number of slices). ***c***, Threshold EPSP slope for evoking PS in absence and presence of picrotoxin (100 μM, PTX) in the same slices after three times of ECS. A significant difference between CNT and ECS was observed only in the absence of PTX (Bonferroni’s test following two-way repeated measure ANOVA; Saline, ** *P* < 0.01, interaction [drug treatment × ECS]; *F*_(1,17)_ = 8.14, *P* = 0.011, n represents the number of slices). **d**, Left: A diagram showing the electrode arrangement for recording evoked postsynaptic currents. Center: EPSCs and monosynaptic IPSCs recorded in the same GCs. Scale bars: 20 ms, 100 pA. Right: Reduced IPSC/EPSC ratios after three times of ECS (*t*_(27)_ = 3.375, ** *P* = 0.0023, n represents the number of cells). **e**, Threshold EPSP slopes for evoking PS at 14 days after 11 times of ECS (*t*_(13)_ = 2.543, * *P* = 0.0245, n represents number of slices). **f**, Effect of CPP (1 or 5 treatments at 20 mg/kg) injected after each ECS as indicated on frequency facilitation at 14 days after 11 times of ECS (1 treatment: *t*_(11)_ = 2.73, * *P* = 0.0196, 5 treatments: *t*_(8)_ = 4.293, ** *P* = 0.0026). The number (n) is shown in the graph. Data are presented as means ± SEM

Although repeated ECS increased somatic excitability of GCs (Fig. 3b), this effect was relatively small. Since neuronal excitation depends on synaptic excitation/inhibition (E/I) balance as well, we examined the effect of ECS on excitation of GCs evoked by stimulation of synaptic inputs. The medial perforant path (MPP) input to GCs was stimulated, and evoked EPSPs and GC population spikes were recorded in the GC layer (Fig. 8a). In ECS-treated mice, the coupling between EPSPs and population spikes was significantly enhanced (Fig. 8b). The GABA_A_ receptor antagonist picrotoxin abolished the difference in the EPSP-spike coupling between control and ECS-treated mice (Fig. 8c). Thus, the enhanced synaptic excitability observed after ECS is largely due to a change in synaptic E/I balance toward excitation. Using whole-cell recording, we confirmed that the amplitude of the evoked inhibitory postsynaptic currents (IPSCs), relative to the excitatory postsynaptic currents (EPSCs), was significantly reduced in ECS-treated GCs (Fig. 8d). The EPSP-spike coupling remained enhanced at 14 days after 11 times of ECS (Fig. 8e). These results demonstrated that ECS causes a sustained increase in synaptic excitability in the GCs.

This continuous enhancement of synaptic activation in GCs through, and also beyond, the period of ECS treatments suggests a possible involvement of endogenous neuronal excitation during the non-stimulated period in the establishment of the lasting change in the maturation-related phenotype of GCs. To test this possibility, we examined the effect of an NMDAR antagonist applied during the inter-stimulus period. The NMDAR antagonist, 3-(2-carboxypiperazin-4-yl)propyl-1-phosphonic acid (CPP), was administered during the latter half of ECS treatments. To minimize the effect of CPP on NMDAR activation that was directly induced by ECS, CPP was injected shortly after each ECS. This CPP treatment significantly reversed the reduction of frequency facilitation at 14 days after 11 times of ECS (Fig. 8f). Even a single CPP injection after the last ECS was effective in reversing the reduction of frequency facilitation (Fig. 8f). These results suggest that the endogenous neuronal activity contributes to the lasting change in the maturation-related phenotype of GCs via NMDAR activation.

## Discussion

**Fig. 9:**
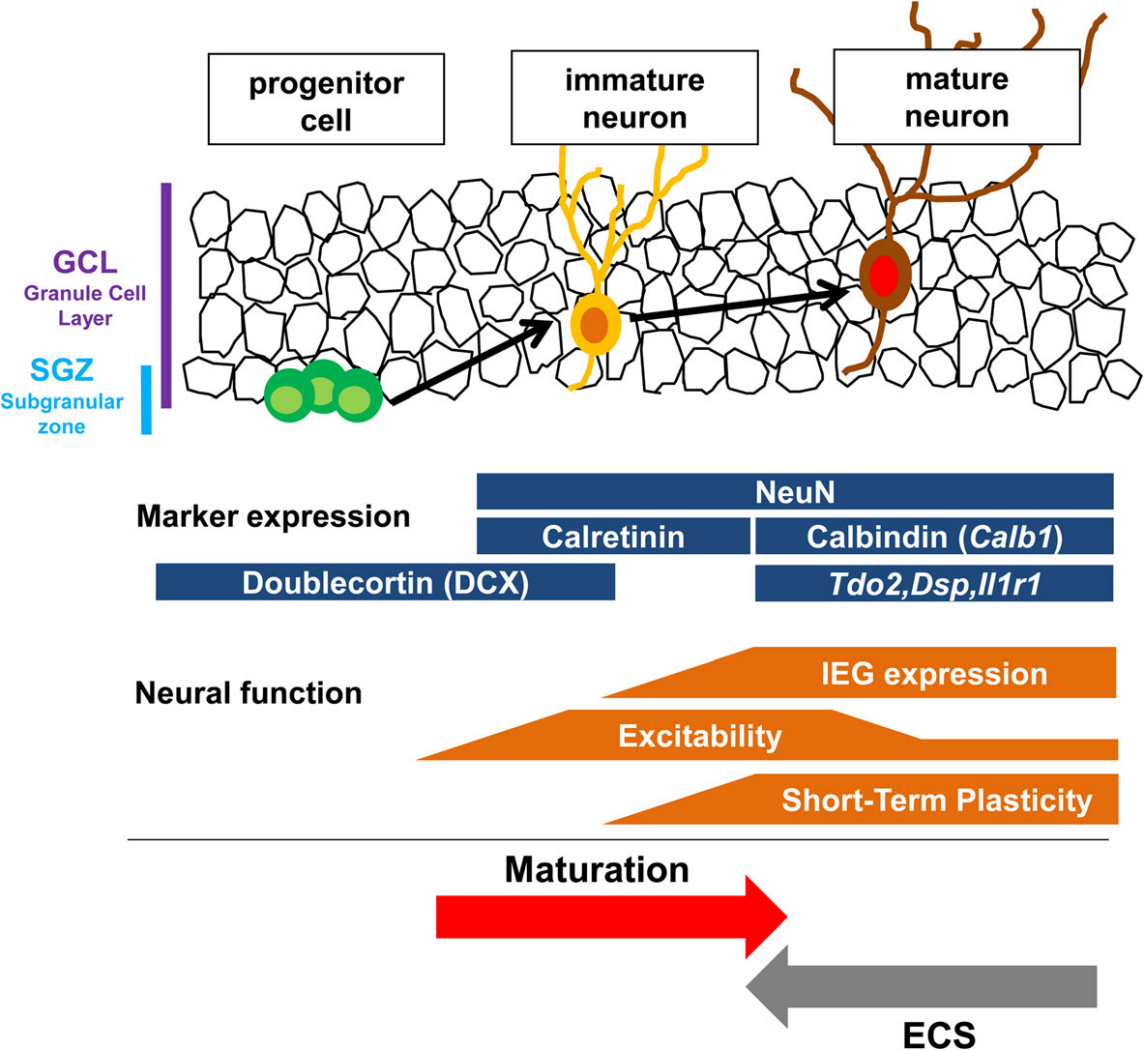
Models of changes in maturation-related phenotypes of the adult GCs by ECS. Developmental stages of GCs in the DG can be identified by stage marker expression and neuronal function

Accumulating evidence suggests modifications of the maturation status of brain neurons as a novel cellular basis of treatments for psychiatric disorders [7, 8, 10–12, 20]. Here we demonstrate that ECS profoundly changes biochemical and physiological features of the mature dentate GCs: ECS reduced the levels of mature neuronal markers, reduced activity-dependent neuronal responsiveness, and increased somatic excitability in the dentate GCs, and also reduced frequency facilitation of MF EPSPs. Since the magnitude of MF frequency facilitation correlates well with the expression level of the mature GC marker *Calb1*, it can be a good physiological index of the functional maturity of GCs [7]. After repeated ECS treatments, the magnitude of facilitation was comparable to that of control mice around postnatal day 10 [7]. The rapid reduction in the expression of mature GC markers after a single ECS and the lack of effects of X-ray irradiation on the reduction of *Calb1* expression indicated that such “demature” GCs are distinct from newly generated immature neurons. Immature GCs generally exhibit higher input resistance than mature GCs, due to their small cell size as indicated by small capacitance and also due to a difference in the resting current density. In the present study, ECS-treated GCs showed normal input resistance and membrane capacitance (data not shown), suggesting no significant change in the cell size. At the later stage of GC maturation, the input resistance is inversely proportional to the membrane capacitance [23], suggesting that the cell size is the critical determinant of the input resistance at late maturation of GCs. Therefore, a plausible interpretation of our observations is that ECS can apparently reverse some of the biochemical and physiological alterations characterizing the late-stage maturation of GCs without changing the overall morphology of them (Fig. 9).

We have previously shown that the SSRI antidepressant fluoxetine can induce GC dematuration [7, 8, 10, 20]. The phenotype of ECS-treated GCs investigated here resembled those of the SSRI-treated demature GCs, as exemplified by similar comprehensive gene expression profiles in the DG. Since ECS has been considered to be an animal model of the ECT used for depression, our results suggest that dematuration of neurons in the DG could be a common cellular basis for pharmacological and physical antidepressant treatments. One of the common functional features of the GCs found in SSRI- and ECS-treated mice is increased somatic excitability. In addition, ECS-treated GCs show alteration of the synaptic E/I balance toward excitation. It has been suggested that neuronal excitation or the E/I balance in the DG is perturbed by chronic stress [27–29]. It is possible that GC dematuration restores neuronal activity in the DG of individuals in a depressed state by enhancing the synaptic and/or intrinsic excitability. Clinically, ECT has fast-acting antidepressant effects and is effective in most of patients who are resistant to monoaminergic antidepressant drugs. Consistent with this clinical setting, the phenotypic change in GCs by ECS emerges rapidly and does not require the serotonergic system. Although the magnitude of dematuration, as assessed by the reduction of frequency facilitation, is on average similar between ECS and SSRI, the effect of ECS is much less variable than that of SSRIs (compare Fig. 3g in this study and Fig. 3b in [7]). In SSRI-treated mice, the excessive change in the maturation-related phenotype was accompanied by a marked increase in day-to-day fluctuation of home-cage activity levels that resembled an antidepressant-induced rapid change in mood [8]. In addition, α-CaMKII hetero KO mice show profound immaturity of the DG and infradian oscillation of home-cage activity levels [2]. Thus, the invariable effect of ECS may be related to a relatively low risk for switching to mania in patients with bipolar disorder who are treated with ECT [30]. While ECT rapidly alleviates acute episodes of depression, relapse rates are relatively high after discontinuation of the therapy [31, 32]. Based on our model, increased neuronal activity and/or augmentation of NMDAR activation may help to prevent relapse of depressive symptoms after discontinuation of ECT.

We demonstrated that the synaptic E/I balance is changed toward excitation in the ECS-treated GCs, and that the excitability of the ECS-treated GCs is continuously enhanced. Enhanced GABAergic inhibition by diazepam after the period of ECS treatments partially reversed the altered phenotype of GCs to the initial matured state (Fig. 7e), suggesting that endogenous neuronal activity is required for lasting maintenance of the demature state of GCs. Since block of NMDAR activation during the non-stimulated period also attenuated the long-lasting effect of ECS on the phenotype of GCs, it is likely that the enhanced synaptic excitation of these cells supports their demature state via NMDAR activation. Recent studies including our own have suggested that both changes in the E/I balance and aberrant neuronal maturation are involved in the pathophysiological basis of schizophrenia and related neuropsychiatric disorders [2, 5, 26, 33–36]. It is possible that defects in neuronal maturation could alter the circuit E/I balance, and a change in the E/I balance toward over-excitation may anchor the aberrant state of maturation in these disorders. Enhanced inhibition may benefit this type of psychiatric disorder, not only by correcting the circuit E/I balance, but also by allowing proper maturation of neurons in the circuit. Indeed, enhancing GABAergic inhibition has been suggested as a treatment strategy for schizophrenia [37]. Further investigation of the regulation of neuronal maturation by neuronal activity would facilitate understanding of the heterogeneous pathogenic and pathophysiological mechanisms underlying neurodevelopmental psychiatric disorders.

## Methods

### Animals

Male C57BL/6J or C57BL/6N mice, 8 weeks of age, were purchased from Japan SLC or Charles River Japan. The 5-HT4 receptor mutant mice (strain name: B6.129P2-Htr4 < tm1Dgen>/J > backcrossed to the C57BL/6J background more than 10 times were purchased from the Jackson Laboratory. Male and female homozygous mutant mice and their wild-type littermates from heterozygous mating were used in this study. Mice were pair-housed and maintained under standard conditions with a 12-h light/dark cycle and *ad libitum* access to food and water unless otherwise stated. All mice were habituated for over one week before experimental procedures.

### Electroconvulsive stimulation

Bilateral ECS (current, 25 mA; shock duration, 1 s; frequency, 100 pulse/s; pulse width, 0.5 msec) was administered via moistened, spring-loaded ear-clip electrodes with a pulse generator (ECT Unit; Ugo Basile) to mice that were anesthetized with isoflurane (1.5 to 2%) in order to avoid sudden unexpected death associated with seizures. In repeated treatments, ECS was administered 4 times a week for up to 3 weeks. The sham-treated animals were handled in an identical manner to the ECS-treated animals without the administration of shock. In cranial irradiation experiments, ECS was started 14 day after irradiation. Cranial X-ray irradiation was administered as previously described [20]. X-ray (Rigaku Radiofrex 350 X-ray generator) at a dose of 10 Gy was delivered at a dose rate of 0.74 Gy/min.

### Drug treatments

Fluoxetine hydrochloride (LKT Labs) was dissolved in drinking water and orally applied at a dose of 22 mg/kg/day for 4 weeks as described previously [7]. The fluoxetine solutions were prepared every day, and daily fluoxetine intake was determined for individual mice on the basis of the water consumption during the preceding 24 h and the body weight measured every other day. Saccharin (0.2%) was included in the fluoxetine solution to keep water intake comparable to the baseline. Diazepam (Wako) was dissolved in dimethyl sulfoxide (DMSO) at 10 to 20 mg/ml, diluted in the drinking water, and administered at 5 to 10 mg/kg/day. D-AP5 (Tocris Bioscience) was dissolved in saline and intracerebroventricularly administered (1 μg/mouse) under anesthesia with pentobarbital (50 mg/kg) 20 min prior to ECS. Cycloheximide (Santa Cruz) was dissolved in saline and intraperitoneally administered at a dose of 200 mg/kg 30 min before ECS. For deletion of serotonergic neurons, 5,7-DHT (Sigma Aldrich) was dissolved in saline containing 0.1% ascorbic acid and was intracerebroventricularly administered (200 μg/mouse) under anesthesia with pentobarbital (50 mg/kg) 30 min after intraperitoneal injection of desipramine (25 mg/kg; Sigma Aldrich). The deletion of serotonergic neurons 1 week after the injection was confirmed in equally conditioned mice by immunohistochemical analysis using anti 5-HT antibody (Immunostar 20080, diluted 1:10000). CPP (Sigma-Aldrich) was dissolved in saline and intraperitoneally injected at a dose of 20 mg/kg shortly after ECS procedures.

### Real time PCR

Mice were decapitated at the time indicated in figure legends and the DG of the hippocampus was dissected under a stereoscopic microscope. Total RNA was extracted from the isolated DG by using an RNeasy micro kit (Qiagen) or Reliaprep RNA Cell Miniprep System (Promega), and subjected to the reverse transcription reaction with a Superscript VILO (Invitrogen), followed by real time PCR with a LightCycler (Roche Applied Science) using Fast Start DNA Master SYBR Green I. Crossing point values were acquired by using the second derivative maximum method. The expression level of each gene was quantified using external standardized dilutions. Relative expression levels of target genes between samples were normalized to that of 18S rRNA. The specificity of each primer set was confirmed by checking the product size by gel electrophoresis. Primer sequences for each gene are shown in Table 5.

**Table 5:** Primer sequences used for real-time RT-PCR

Gene	Forward	Reverse
*Calb1*	5′- tctggcttcatttcgacgctg-3′	5′- acaaaggatttcatttccggtga-3′
*Tdo2*	5′- atgagtgggtgcccgtttg-3′	5′- ggctctgtttacaccagtttgag-3′
*Dsp*	5′- gctgaagaacactctagccca-3′	5′- actgctgtttcctctgagaca-3′
*Il1r1*	5′- gtgctactggggctcatttgt-3′	5′- ggagtaagaggacacttgcgaat-3′
*Fos*	5′- ctgtcaacacacaggactttt-3′	5′- aggagatagctgctctactttg-3′
*18S rRNA*	5′- gaggccctgtaattggaatgag-3′	5′- gcagcaactttaatatacgctattgg-3′

### Immunohistochemistry

Mice were perfused with saline and 4% paraformaldehyde in 0.1 M phosphate buffer, pH 7.4. The brains were dissected out and postfixed in the same fixative at 4 °C for 24 h. After immersion in 0.1 M phosphate buffer containing 20% sucrose at 4 °C overnight, the brains were rapidly frozen at -80 °C and sectioned using a cryostat at 30 μm thickness. The free-floating sections were first incubated with 10% normal equine serum in PBS containing 0.3% Triton X-100 for 1 h at room temperature and subsequently incubated with mouse anti-calbindin-D-28 K monoclonal antibody (Sigma Aldrich, C9848, diluted 1:3000), mouse anti-calretinin monoclonal antibody (Millipore, MAB 1568, diluted 1:3000), goat anti-doublecortin polyclonal antibody (Santa Cruz, sc-8066, diluted 1:500), rabbit anti-c-Fos polyclonal antibody (Calbiochem, PC38, diluted 1:2000), or mouse anti-NeuN antibody (Millipore, MAB377, diluted 1:500) overnight at 4 °C. After washing three times with PBS containing 0.3% Triton X-100, the sections were incubated with secondary antibody conjugated with AlexaFluor488 or AlexaFluor555 (Molecular Probes). After washing, the sections were mounted on slides and observed with a fluorescent microscope (Biozero 8000, Keyence). For some sections of doublecortin immunostaining, biotinylated horse anti-goat IgG (Vector) was used as a secondary antibody, followed by incubation with ABC Vectastain Kit (Vector). Antigen detection was performed with 3,3′ -diaminobenzidine staining.

### Electrophysiological analysis

Mice were singly housed in the institutional standard condition (14:10 light/dark cycle; lights on at 6:00 A.M. through 8:00 P.M.). Mice were decapitated under deep halothane anesthesia 24 h after the last ECS unless otherwise stated. Both hippocampi were isolated, and transverse hippocampal slices (380 μm) were cut using a tissue slicer. Electrophysiological recordings were performed as described [7]. Recordings were made in a submersion-type chamber maintained at 27.0–27.5 °C and superfused at 2 ml/min with saline composed of (in mM): NaCl, 125; KCl, 2.5; NaH_2_PO_4_, 1.0; NaHCO_3_, 26.2; glucose, 11; CaCl_2_, 2.5; MgCl_2_, 1.3 (equilibrated with 95% O_2_/5% CO_2_). EPSPs arising from the MF synapses were evoked by stimulating the dentate granule cell layer and recorded from the stratum lucidum of CA3 using a glass pipette filled with 2 M NaCl. The amplitude of field EPSPs was measured on analysis as described [38]. A criterion used to identify the MF input was more than 85% block of EPSP by an agonist of group II metabotropic glutamate receptors, (2S,2′R,3′R)-2-(2′,3′-dicarboxycyclopropyl)glycine (DCG-IV, 1 μM). Single electrical stimulation was delivered at a frequency of 0.05 Hz unless otherwise specified. For recording EPSPs and population spikes evoked by activation of the MPP input to GCs, the recording electrode was placed in the granule cell layer, and electrical stimulation was delivered every 30 s via the electrode placed in the middle third of the molecular layer. The initial slope of EPSPs was measured on analysis. The amplitude of the population spike was measured as the difference between the negative peak and the average of two positive peaks. Whole-cell current-clamp recordings were made from GCs with a pipette filled with a solution composed of (in mM) potassium gluconate 140, HEPES 20, NaCl 8, MgATP 2, Na_2_GTP 0.3, EGTA 0.05 (pH adjusted to 7.3 with KOH). The recording pipette was placed in the middle third of the granule cell layer. Hyperpolarizing currents (6 - 16 pA, 400 ms) were injected through the recording pipette to measure the input resistance. Depolarizing currents (400 ms) with increasing intensity by 10 pA steps were injected to measure the threshold current intensity to evoke action potentials. Whole-cell voltage-clamp recordings were made from GCs with a pipette filled with a solution composed of (in mM) cesium gluconate 140, HEPES 20, NaCl 8, EGTA 0.1, MgATP 2, Na_2_GTP 0.3 (pH adjusted to 7.3 with CsOH). EPSCs evoked by MPP stimulation were recorded at the reversal potential (-70 mV) for IPSCs. The amplitude of EPSC was adjusted around 100 pA. Monosynaptic IPSCs were then recorded in the same cells at +1 mV in the presence of 6-cyano-7-nitroquinoxaline-2,3-dione (CNQX, 10 μM) and D-AP5 (25 μM). The liquid junction potential was corrected in these recordings. All recordings were made using a Multiclamp 700B amplifier (Molecular Devices, Sunnyvale, CA, USA), filtered at 2 kHz and stored in a personal computer via an interface (digitized at 5 - 10 kHz). DCG-IV and CNQX were purchased from Tocris Bioscience (Bristol, UK). Picrotoxin was from Wako Pure Chemical Industries, Ltd (Osaka, Japan).

### In situ hybridization

In situ hybridization was performed using a digoxigenin (DIG)-labeled riboprobe as described [39]. *Calb1* cDNA template probe was cloned by PCR with gene-specific primers (Forward; 5′-actgaccacagcggcttc-3′, Reverse; 5′-agaggcagaagcccatcc-3′, Product Length; 927 bp), verified by sequencing, and used to produce a labeled riboprobe with the RNA Labeling kit (Roche). The brains were removed from mouse skulls at 6 h after single ECS and rapidly frozen on dry ice. None of the sense probes yielded any signal.

### Microarray analysis

For comparison of gene expression profiles between single ECS-treated and repeated ECS-treated DG, the same RNA samples as in Fig. 2a and b were used. Dematured DG and control samples were collected as follows. Mice were decapitated at 24 h after the 11 times of ECS or 4-week treatment of fluoxetine at a dose of 22 mg/kg. The hippocampal slices were prepared as described above. The dentate gyrus was dissected from some of them, and the remaining slices were used for electrophysiological analyses. Frequency facilitation at the MF-CA3 synapse was measured in each mouse, and the DG samples from fluoxetine-treated mice that exhibited small frequency facilitation were used as dematured DG (*n* = 3). Reduced facilitation was also confirmed in ECS-treated mice. The control DG samples were collected from vehicle- or sham-treated mice. Total RNA was extracted by using an RNeasy micro kit (Qiagen) and the samples of the same groups were put together. From each group, 100 ng of total RNA was amplified with 3′IVT Express kit (Affymetrix, Inc.). All samples were hybridized to the GeneChip mouse genome 430A 2.0 array (Affymetrix, Inc.), and the microarray suite 5.0 of the Affymetrix gene chip operating software was used for the analysis of the GeneChip data. For each transcript represented on the array, the statistical expression algorithm computes detection (present or absent), signal intensity, change (increase or decrease), and change *p*-value. For the comparison in the DG between repeated ECS- and chronic fluoxetine-treatment, if the average signal intensity in all of the conditions (ECS, sham, fluoxetine, control) was lower than 50, the genes were excluded from further analysis. Hippocampal gene expression data in Shn-2 KO mice and αCaMKII hetero KO mice was obtained from [26] and [2], respectively. Correlations were examined with Spearman correlation coefficients. Gene ontology analysis was performed using DAVID Bioinformatics Resources 6.8 (National Institute of Allergy and Infectious Diseases).

### Immunoblot

The brains were removed at 24 h after the last ECS and the DG of the hippocampus was dissected under a stereoscopic microscope. The isolated DG was sonicated in protein lysis buffer containing protease inhibitor cocktail (Nacalai tesque) on ice and centrifuged at 20,000 *g* for 10 min at 4 °C. Supernatants containing 10 μg of proteins were separated on 12% SDS-polyacrylamide gel by electrophoresis and transferred onto polyvinyl difluoride membrane. The membrane was first blocked with Blocking One (Nacalai tesque) for 1 h at room temperature and then incubated with mouse anti-calbindin-D-28 K monoclonal antibody (diluted 1:3000) or anti-PSD-95 monoclonal antibody (BD bioscience, BD610496, diluted 1:500) at 4 °C overnight. After washing, the membrane was incubated with horseradish peroxidase conjugated secondary antibody (Jackson) for 1 h at room temperature, and bands were visualized with the ECL reagent (GE Healthcare). The same membrane was then stripped, and detection of β-actin was performed using mouse anti-β-actin monoclonal antibody (Millipore, MAB1501R, diluted 1:3000) in the same way. The signal intensity of calbindin was calculated by LAS-3000 (FujiFilm) and normalized to that of β-actin.

### Statistics

All data are presented as means ± SEM. Experiments with two groups were compared with unpaired two-tailed Student’s *t* test unless otherwise specified, and experiments with more than two groups were subjected to one-way ANOVA, followed by the Dunnett’s test or the Tukey’s test. Interaction between subgroups was compared with two-way ANOVA, followed by the Bonferroni’s test. Statistical significance was set at *P* < 0.05. The number of data “n” represents the number of mice used unless otherwise specified.

## Additional file

Additional file 1: Table S1. Functional classification of the genes that showed reduced responsiveness 1 h after repeated ECS treatments compared with a single ECS on the basis of the gene ontology (GO) terms. **Table S2.** Functional classification of the genes that showed similar responsiveness 1 h after repeated ECS treatments compared with a single ECS on the basis of the GO terms. **Table S3.** Functional classification of the genes that showed significantly increased expression change by only ECS treatment (317 gene probes) on the basis of the gene ontology (GO) terms. **Table S4.** Functional classification of the genes that showed significantly decreased expression change by only ECS treatment (444 gene probes) on the basis of the GO terms. **Table S5.** Functional classification of the genes that showed significantly increased expression change by only SSRI treatment (690 gene probes) on the basis of the GO terms. **Table S6.** Functional classification of the genes that showed significantly decreased expression change by only SSRI treatment (739 gene probes) on the basis of the GO terms. (PDF 169 kb)

## Abbreviations

5,7-DHT: 5,7-dihydroxytryptamine
5-HT4R: serotonin type 4 receptor
CNQX: 6-cyano-7-nitroquinoxaline-2,3-dione
CPP: 3-(2-carboxypiperazin-4-yl)propyl-1-phosphonic acid
D-AP5: D-(-)-2-amino-5-phosphonopentanoic acid
DCG-IV: (2S,2′R,3′R)-2-(2′,3′-dicarboxycyclopropyl)glycine
DG: Dentate gyrus
E/I: Excitation/inhibition
ECS: Electroconvulsive stimulation
ECT: Electroconvulsive therapy
EPSCs: Excitatory postsynaptic currents
EPSPs: Excitatory postsynaptic potentials
GCs: Granule cells
IEGs: Immediate early genes
IPSCs: Inhibitory postsynaptic currents
MFs: Mossy fibers
MPP: Medial perforant path
NMDAR: N-methyl-D-aspartate receptor
SSRI: Selective serotonin reuptake inhibitor
