# Supplementary material for: Rapid and stable changes in maturation-related phenotypes of the adult hippocampal neurons by electroconvulsive treatment
Source: Mol Brain. 2017 Mar 2;10:8. doi: 10.1186/s13041-017-0288-9 (PMC5335812; doi:10.1186/s13041-017-0288-9)
Supplement: Additional file 1: Table S1. — Functional classification of the genes that showed reduced responsiveness 1 h after repeated ECS treatments compared with a single ECS on the basis of the gene ontology (GO) terms. Table S2. Functional classification of the genes that showed similar responsiveness 1 h after repeated ECS treatments compared with a single ECS on the basis of the GO terms. Table S3. Functional classification of the genes that showed significantly increased expression change by only ECS treatment (317 gene probes) on the basis of the gene ontology (GO) terms. Table S4. Functional classification of the genes that showed significantly decreased expression change by only ECS treatment (444 gene probes) on the basis of the GO terms. Table S5. Functional classification of the genes that showed significantly increased expression change by only SSRI treatment (690 gene probes) on the basis of the GO terms. Table S6. Functional classification of the genes that showed significantly decreased expression change by only SSRI treatment (739 gene probes) on the basis of the GO terms. (PDF 169 kb) [file 13041_2017_288_MOESM1_ESM.pdf]

Table S1. Functional classification of the genes that showed reduced responsiveness 1 h after repeated ECS treatment compared with a single ECS on the basis of the gene ontology (GO) terms.

**Reduced responsiveness after repeated ECS treatment**			
Genes increased by a single ECS (195 probes)			
GO number	GO term	No.	P value
GO:0045944	positive regulation of transcription from RNA polymerase II promoter	26	7.94E-08
GO:0007623	circadian rhythm	10	1.15E-07
GO:0006355	regulation of transcription, DNA-templated	40	3.59E-07
GO:0000122	negative regulation of transcription from RNA polymerase II promoter	21	4.79E-07
GO:0035914	skeletal muscle cell differentiation	7	3.33E-06
GO:0043066	negative regulation of apoptotic process	16	2.18E-05
GO:0051591	response to cAMP	6	3.84E-05
GO:0007179	transforming growth factor beta receptor signaling pathway	6	2.44E-04
GO:0034976	response to endoplasmic reticulum stress	6	2.60E-04
GO:0071376	cellular response to corticotropin-releasing hormone stimulus	3	3.29E-04
GO:0048661	positive regulation of smooth muscle cell proliferation	6	3.31E-04
GO:0034097	response to cytokine	6	3.50E-04
GO:0032870	cellular response to hormone stimulus	5	4.13E-04
GO:0001701	in utero embryonic development	10	4.15E-04
GO:0009416	response to light stimulus	4	8.30E-04
Genes decreased by a single ECS (117 probes)			
GO number	GO term	No.	P value
GO:0006355	regulation of transcription, DNA-templated	25	9.15E-04

P-value was calculated by a modified Fisher's exact test. Highly associated gene ontology terms (P < 0.001) were represented.

Table S2. Functional classification of the genes that showed similar responsiveness 1 h after repeated ECS treatment compared with a single ECS on the basis of the gene ontology (GO) terms

**No significant change of responsiveness between a single and repeated ECS**			
Genes increased by a single ECS (122 probes)			
GO number	GO term	No.	P value
GO:0043066	negative regulation of apoptotic process	13	6.10E-05
GO:0008360	regulation of cell shape	7	1.15E-04
GO:0001525	angiogenesis	8	3.42E-04
Genes decreased by a single ECS (933 probes)			
GO number	GO term	No.	P value
GO:0006355	regulation of transcription, DNA-templated	164	2.23E-17
GO:0006351	transcription, DNA-templated	124	3.42E-10
GO:0006974	cellular response to DNA damage stimulus	36	8.72E-06
GO:0006397	mRNA processing	29	3.09E-05
GO:0006281	DNA repair	27	1.63E-04
GO:0032259	methylation	17	6.05E-04

P-value was calculated by a modified Fisher's exact test. Highly associated gene ontology terms ($P < 0.001$) were represented.

Table S3. Functional classification of the genes that showed significantly increased expression change by only ECS treatment (317 gene probes) on the basis of the gene ontology (GO) terms

**Increased change by only ECS-treatment (317 probes)**			
GO number	GO term	No.	P value
GO:0006695	cholesterol biosynthetic process	6	2.01E-05
GO:0016126	sterol biosynthetic process	6	7.80E-05
GO:0046942	carboxylic acid transport	9	1.24E-04
GO:0015849	organic acid transport	9	1.33E-04
GO:0048514	blood vessel morphogenesis	12	2.10E-04
GO:0001568	blood vessel development	13	3.35E-04
GO:0001944	vasculature development	13	4.19E-04
GO:0006694	steroid biosynthetic process	7	6.95E-04

P-value was calculated by a modified Fisher's exact test. Highly associated gene ontology terms (P < 0.001) were represented.

Table S4. Functional classification of the genes that showed significantly decreased expression change by only ECS treatment (444 gene probes) on the basis of the GO terms

**Decreased change by only ECS-treatment (444 probes)**			
GO number	GO term	No.	P value
GO:0006397	mRNA processing	31	3.39E-12
GO:0008380	RNA splicing	23	4.12E-09
GO:0006351	transcription, DNA-templated	75	1.52E-08
GO:0006355	regulation of transcription, DNA-templated	83	9.89E-08
GO:0048025	negative regulation of mRNA splicing, via spliceosome	6	1.51E-04
GO:0006376	mRNA splice site selection	5	1.87E-04

P-value was calculated by a modified Fisher's exact test. Highly associated gene ontology terms (P < 0.001) were represented.

Table S5. Functional classification of the genes that showed significantly increased expression change by only SSRI treatment (690 gene probes) on the basis of the GO terms

**Increased change by only SSRI-treatment (690 probes)**			
GO number	GO term	No.	P value
GO:0001763	morphogenesis of a branching structure	15	4.07E-05
GO:0007242	intracellular signaling cascade	52	5.33E-05
GO:0030029	actin filament-based process	16	4.77E-04
GO:0006909	phagocytosis	8	8.24E-04
GO:0016055	Wnt receptor signaling pathway	13	8.56E-04
GO:0008104	protein localization	41	9.13E-04

P-value was calculated by a modified Fisher's exact test. Highly associated gene ontology terms (P < 0.001) were represented.

Table S6. Functional classification of the genes that showed significantly decreased expression change by only SSRI treatment (739 gene probes) on the basis of the GO terms

**Decreased change by only SSRI-treatment (739 probes)**			
GO number	GO term	No.	P value
GO:0051276	chromosome organization	8	3.80E-04
GO:0006412	translation	29	9.60E-04

P-value was calculated by a modified Fisher's exact test. Highly associated gene ontology terms ($P < 0.001$) were represented.
